# An alternative electrochemical approach for toluene detection with ZnO/MgO/Cr_2_O_3_ nanofibers on a glassy carbon electrode for environmental monitoring†

**DOI:** 10.1039/d0ra08577d

**Published:** 2020-12-17

**Authors:** M. M. Alam, Abdullah M. Asiri, M. T. Uddin, Mohammed M. Rahman, M. A. Islam

**Affiliations:** Department of Chemical Engineering and Polymer Science, Shahjalal University of Science and Technology Sylhet 3100 Bangladesh alam-mahmud@hotmail.com; Department of Chemistry, King Abdulaziz University, Faculty of Science P.O. Box 80203 Jeddah 21589 Saudi Arabia mmrahman@kau.edu.sa; Center of Excellence for Advanced Materials Research (CEAMR), King Abdulaziz University P.O. Box 80203 Jeddah 21589 Saudi Arabia

## Abstract

*In situ* fabrication of a sensitive electrochemical sensor using a wet-chemically prepared ternary ZnO/MgO/Cr_2_O_3_ nanofiber (NF)-decorated glassy carbon electrode (GCE) with Nafion adhesive was the approach of this study. The resultant NFs were characterized by various tools, such as powder X-ray diffraction (XRD), X-ray photoelectron spectroscopy (XPS), field emission scanning electron microscopy (FESEM), energy-dispersive X-ray spectroscopy (EDS), Fourier transform infrared spectroscopy (FTIR), Brunauer–Emmett–Teller (BET) surface area analysis, and ultraviolet–visible spectroscopy (UV/Vis). The analytical parameters of the proposed toluene sensor were characterized as follows: good sensitivity (23.89 μA μM^−1^ cm^−2^), a lower limit of detection (LOD; 95.59 ± 1.5 pM), a limit of quantification (LOQ; 318.63 ± 2.0 pM), efficient response time (18 s), and the dynamic range (LDR) for toluene detection of 0.1 nM–0.01 mM. The real-time application of the sensor is to protect the environmental ecosystem, as well as the public health from the harmful effects of toluene. In an environmental application, the toluene sensor exhibited good reproducibility, robustness, LOD, LOQ, and good reliability, which are discussed in detail and compared to the literature.

## Abstract 2

*In situ* fabrication of a sensitive electrochemical toluene sensor probe using wet-chemically prepared ternary ZnO/MgO/Cr_2_O_3_ nanofiber (NF)-decorated glassy carbon electrode (GCE) with Nafion adhesive was the approach of this study.

## Graphical abstract

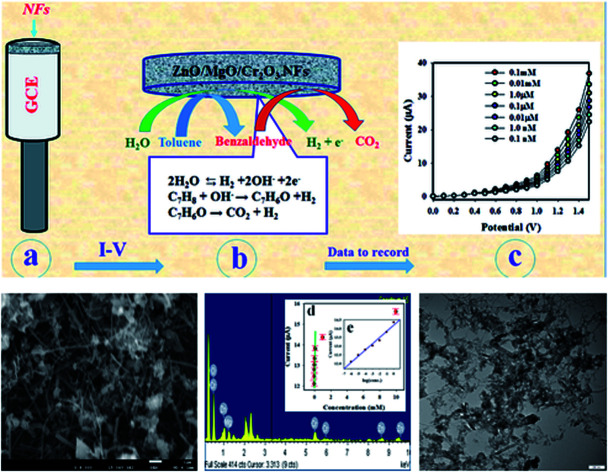

## Introduction

In general, the organic volatile compounds emitted from industrial effluents, automobile exhaust, incineration, and other processes are responsible for the long- and short-term adverse health effects. The health and environmental organizations have designed the maximum contamination limit of these volatile organic compounds in the indoor and outdoor environments.^1^ Among these volatile organic compounds, toluene (C_7_H_8_) is widely used in the chemical industry and is found to be neurotoxin, which is harmful to the human body at low concentrations.^2^ Toluene is used in many applications, such as flame retardants, fuel systems, antifreeze, rust preservatives, metal cleaners, adhesives, glues, nail polish, thinners, varnishes, and paints.^3^ Besides this, toluene is applied as a raw material to produce detergents, perfumes, dyes, inks, plastics, medicines and various chemicals.^4,5^ Due to the toxicity of toluene, the impact on public health and environmental ecosystem is hazardous. The small amount of (less than 200 ppm) toluene inhalation is responsible for numerous illnesses in humans, such as dilated pupils, exhaustion, anxiety, euphoria, weakness, irritation in the eyes and nose, tingling in the skin and dermatitis, lacrimation, confusion, insomnia, and fatigue.^6,7^ As toluene is a flammable and toxic, reliable detection methods are desired to protect the public health and environment.

Of late, the electrochemical sensors using sensing elements (such as semi-conductive metal oxides) are becoming popular in the detection of environmental toxic chemicals.^8–10^ Numerous metal oxides, including Cr_2_O_3_,^11^ ZnO,^12^ SnO_2_,^13^ WO_3_,^14^ α-Fe_2_O_3_,^15^ Co_3_O_4_,^16^ In_2_O_3_,^17^ and the hybrid composite of these metal oxides^18,19^ have been investigated as sensing substrates of toluene. However, most research studies have been executed to detect toluene in the gaseous phase. As toluene has a wide range of applications, there is a great risk of contamination of the aqueous environment. Therefore, this study was approached to develop a toluene sensor with semi-conductive metal oxide nanomaterials, which are capable of the detection of the toluene target in an aqueous medium. The selectivity of the traditional sensor is a great challenge, because similar elements exhibit multi-selectivity. To overcome this drawback, two or multi-metal oxides are utilized as the sensing mediator in this modern research approach. Also, a change in the interfacial resistance due to the formation of a heterojunction resulted in the improved performance of the sensors.^20^ It has been reported previously that the binary ZnO–SnO_2_ NFs,^21^ ZnO–Cr_2_O_3_ NRs,^22^ and ZnO–In_2_O_3_ NFs^23^ reliably detect ethanol, trimethylamine, and trimethylamine, respectively. Recently, a few research studies based on the composite metal oxides have been reported to reliably detect toluene in an aqueous medium. So far, there still needs to be further improvements to develop composite metal oxide-based sensors. Among various metal oxides, ZnO is a semi-conductor with advantageous properties, such as 3.37 eV optical band gap, 60 meV free-exciton binding energy, 300 cm^−1^ optical gain, mechanical and thermal stability.^24^ Therefore, considering its ferroelectric, piezoelectric, optical, electrical and magnetic properties, ZnO is a strong candidate to use in the advanced electrochemical device application.^25^ It has been reported that MgO is another potential nanomaterial (metal oxides), and is capable of improving the efficiency of the solar cell.^26^ Besides this, Cr_2_O_3_ is an efficient metal oxide used as a gas-sensing element.^27^

In this study, ZnO/MgO/Cr_2_O_3_ NFs have been considered for its favorable structural, optical and physicochemical properties in terms of its permeability, high porosity, high-stability, and large-active surface area, which are dependent on the reactant precursors (chromium nitrate, zinc nitrate, and magnesium sulphate), and the morphology used in the synthesis of ZnO/MgO/Cr_2_O_3_ NFs at low temperature in the alkaline phase. The nanofibers of ZnO/MgO/Cr_2_O_3_ were prepared using the co-precipitation (wet-chemical) method, applying NH_4_OH as the precipitating agent. This wet-chemical preparation has effectual advantages, such as high-porosity, one-step reaction, ease of preparation, precious temperature controlling and facile handling facilities. The resulting properties of the ZnO/MgO/Cr_2_O_3_ NFs (chemical, electrical, optical, and structural morphology) have a great significance in terms of the scientific aspect compared to the un-doped nanomaterials. The conductivity of this ternary ZnO/MgO/Cr_2_O_3_ NFs enhances its non-stoichiometric oxygen vacancies. The energy associated with oxygen vacancies and metal interstitials in the doped-semiconductors is low, and it generated the elevated conductivity of the ZnO/MgO/Cr_2_O_3_ NFs compared to other un-doped semi-conductors. Besides this, the ZnO/MgO/Cr_2_O_3_ NFs have attracted significant research interest owing to their probable applications in the fabrication of a biochemical detector, electron-field emission sources, hybrid-composites, and sensor devices. Moreover, due to the high reactive inter-surface area of the ZnO/MgO/Cr_2_O_3_ NFs on GCE, it possesses the high conductivity, as well as current density in the electrochemical analysis.^28–30^

Therefore, this approach involved the fabrication of a selective toluene sensor applying ternary ZnO/MgO/Cr_2_O_3_ NFs on GCE. The wet-chemically prepared ZnO/MgO/Cr_2_O_3_ NFs were decorated on GCE using Nafion adhesive as a thin film, resulting in the proposed toluene sensor. The fabricated toluene sensor exhibited good performances in terms of its analytical parameters, such as good sensitivity, extended dynamic range (LDR) for toluene detection, the significant lower limit for detection (LOD), efficient response time, precious reproducibility and long-term performing ability in the electrochemical sensing performance of toluene in the buffer phase. To the best of our knowledge, this is the first time that the synthesized ternary ZnO/MgO/Cr_2_O_3_ NFs were applied onto GCE to detect unsafe toluene levels by an electrochemical approach. This approach to the development of an electrochemical sensor for the reliable detection of environmentally unsafe toxic chemicals should be a great advantage in the environmental sector.

## Experimental section

### Chemicals and reagents

Analytical grade inorganic salts, such as Cr(NO_3_)_3_·9H_2_O, MgSO_4_·7H_2_O, and Zn(NO_3_)_2_·6H_2_O, were purchased from the Sigma-Aldrich company (USA), and used directly to prepare ZnO/MgO/Cr_2_O_3_ NFs. To perform this study, the toxic chemicals, including 2-acetylpyridin, M-xylol, 3-methylaniline, zimtaldehyde, 1,4-dioxane, toluene, chlorobenzene, hydroquinone, and paracetamol, were also obtained from Sigma-Aldrich. Besides this, concentrated ammonia, the Nafion adhesive, mono- and disodium phosphate were supplied by Merck Germany to complete this study.

### Synthesis of ZnO/MgO/Cr_2_O_3_ nanofibers by wet-chemical method

The co-precipitation method was used to prepare the nanostructure materials of the doped/un-doped metal oxides with distinct surface-sensitive morphological structure. The ZnO/MgO/Cr_2_O_3_ NFs were prepared to apply this wet-chemical method in the high alkaline phase. This process consists of three sequential parts: (i) co-precipitation of the metal ions in the form of metal hydroxides; (ii) separation of the obtained metal hydroxides, followed by drying in an oven; and (iii) calcination of the metal hydroxides, which was performed in a muffle furnace under pure oxygen flow. Following these procedures, 0.1 M solutions of MgSO_4_·7H_2_O, Zn(NO_3_)_2_·6H_2_O, and Cr(NO_3_)_3_·9H_2_O were prepared in three separate 100.0 mL volumetric flasks using de-ionized water. After that, a 50.0 mL aliquot of the solution from each volumetric flask was taken into a 250.0 mL beaker, and heated at 80 °C on a hot-plate with continuous magnetic stirring. Subsequently, the pH of the mixture was maintained at 10.5 by adding 0.1 M NH_4_OH solution drop-wise. It has been considered that metal ions are co-precipitated quantitatively in the form of metal hydroxides at pH 10.5. After that, the obtained white precipitate of the metal hydroxides was filtrated and washed with water successively to remove the impurities and unreacted metal ions (if any). Finally, the obtained crystal mass was dried at 120 °C in an oven overnight. The probable reaction scheme is as follows:i
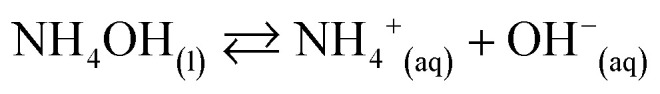

ii Zn(NO_3_)_2(s)_ → Zn^2+^_(aq)_ + 2NO_3_^−^_(aq)_

iii MgSO_4(s)_ → Mg^2+^_(aq)_ + SO_4_^−^_(aq)_

iv Cr_2_(NO_3_)_3(s)_ → 2Cr^3+^_(aq)_ + 3NO_3_^−^_(aq)_

v
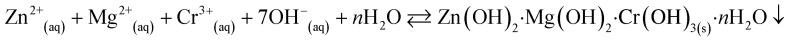


The precipitation of the metal hydroxide depends on the solubility-product-constant *K*_sp_ [*K*_sp_ = 3 × 10^−27^ for Zn(OH)_2_, *K*_sp_ = 5.61 × 10^−12^ for Mg(OH)_2_, and *K*_sp_ = 6.3 × 10^−31^ for Cr(OH)_3_].^31^ With the dropwise addition of NH_4_OH into a beaker, the OH^−^ concentration in the solution was increased gradually and the pH increased. As the *K*_sp_ value of Cr(OH)_3_ is lower compared to other existing metal hydroxides, the precipitation was initiated first and the small nuclei of crystallites were formed to generate the crystal formation. The resulting crystallites of Cr(OH)_3_ were aggregated together to form a large crystallite. As the pH of the solution was continuously increased gradually, the 2nd lowest *K*_sp_ value of Zn(OH)_2_ also began to precipitate, and was followed by the adsorption on the crystallites of Cr_2_(OH)_3_. Subsequently, the 3rd Mg(OH)_2_ with the highest *K*_sp_ value was precipitated similarly. The resultant crystals of the nanomaterials were separated from the aqueous phase, and washed successively with deionized water. Then, the washed nanocrystals of the obtained metal hydroxides were dried in an oven overnight at 120 °C. After that, the dry nanocrystals were ground with a pestle and mortar, and followed by calcination at 500 °C for 6 hours in the muffle furnace. During the calcination process, the metal hydroxides were oxidized to metal oxides in the presence of flowing oxygen gas. The probable reaction involved in the muffle furnace is as follows:

vi Zn(OH)_2_·Mg(OH)_2_·Cr(OH)_3(s)_ + ^3^/_2_O_2_ → ZnO·MgO·Cr_2_O_3_ + ^7^/_2_H_2_O

### Instrumentation

The investigation of the binding energy and ionization states of the existing atoms of the ZnO/MgO/Cr_2_O_3_ NFs were evaluated by XPS analysis performed on a K-α1 spectrometer (Thermo scientific, K-α1 1066) with an excitation radiation source (A1 Kα1, beam spot size = 300.0 μm, pass energy = 200.0 eV, pressure ∼ 10–8 torr). The photosensitivity and existing functional groups of the synthesized ZnO/MgO/Cr_2_O_3_ NFs were evaluated by the application of UV-Vis (Thermo Scientific) and FTIR (Thermo Scientific NICOLET iS50, Madison, WI, USA) spectroscopic analysis. The structural morphology and elemental compositions (shape, size, and structure) of the synthesized ZnO/MgO/Cr_2_O_3_ NFs were inspected by FESEM (JEOL, JSM-7600F, Japan) furnished with EDS. The phase crystallinity of the nanomaterial is an important measurable characteristic, and it is able to determine the unit cell dimension. Therefore, the phase crystallinity was studied by powder X-ray diffraction. The N_2_ adsorption–desorption isotherms were carried out by the 3Flex analyzer (Micromeritics, USA) at 77.0 K. The specific surface area (SBET) was calculated using multi-point adsorption data from the linear segment of the N_2_ adsorption isotherms using BET theory. Finally, the electrochemical detection was performed by a Keithley electrometer (USA).

### Fabrication of working electrode with NFs

The working electrode of the proposed sensor is the dominating part, and was fabricated by modification of a GCE with ZnO/MgO/Cr_2_O_3_ NFs with the help of the Nafion adhesive. For the fabrication of this electrode, a slurry of NFs in ethanol was deposited on the GCE with a surface area of 0.0316 cm^2^ to form a layer of thin film. Then, it was kept at the ambient conditions of the laboratory to dry completely. To enhance the working duration of the electrode, a few drops of Nafion adhesive was put on the surface of the NFs layer on the GCE to boost its stability. After that, the modified GCE was kept in an oven at 35.0 °C for an adequate amount of time to dry it again. An electrochemical cell (chemical sensor) was assembled by connecting the ZnO/MgO/Cr_2_O_3_ NFs/GCE and Pt-wire through the Keithley electrometer to perform as the working and counter electrodes, respectively. The toluene solution at a concentration range of 0.1 nM to 0.1 mM was prepared in deionized water, and applied as the targeted analyte for electrochemical characterization of the sensor. A plot showing the current *versus* concentration of toluene was explored and denoted as a calibration curve, which was found to be linear and identified by regression coefficient *R*^2^. The slope of the calibration-curve and surface area of GCE were used to calculate the sensor sensitivity. By marking a linear segment on the calibration-curve, the dynamic range of detection (LDR) was obtained. The lower limit (LOD) and quantification limit (LOQ) for toluene detection were calculated using a signal/noise (S/N) ratio of 3. During the electrochemical analysis, the buffer solution in the investigating beaker was kept as 10.0 mL as a constant. It should be mentioned that the Keithley electrometer (USA) is a simple two-electrode system device.

## Result and discussion

### XPS analysis of the ZnO/MgO/Cr_2_O_3_ NFs

**Fig. 1:**
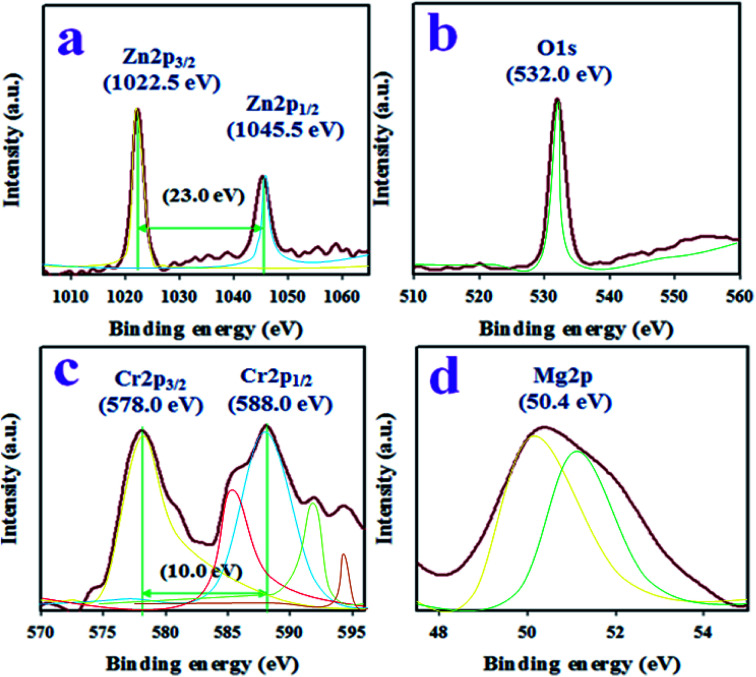
The XPS investigation to evaluate the ionization states and binding energy of the atoms in ZnO/MgO/Cr_2_O_3_ NFs. (a) The XPS spectra of the Zn 2p level orbital, (b) O 1s orbital, (c) core level Cr 2p orbital, and (d) Mg 2p orbital.

The binding energy and ionization states of the existing atoms in the prepared ZnO/MgO/Cr_2_O_3_ NFs were investigated by applying XPS, as illustrated in Fig. 1. As seen in Fig. 1(a), the Zn 2p level orbital is split into two symmetric spin orbitals positioned at 1022.5 and 1045.5 eV, corresponding to Zn 2p_3/2_ and Zn 2p_1/2_, respectively. The spin energy separation between the Zn 2p_3/2_ and Zn 2p_1/2_ orbitals is 23.0 eV, which is recognized for the Zn^2+^ ionization state in the ZnO/MgO/Cr_2_O_3_ NFs, as confirmed by previously reported articles.^31–33^ The resultant XPS peak is fitted with the deconvolution data of Zn^2+^, as shown in Fig. 1(a). Besides this, the O 1s XPS spectra (as in Fig. 1(b)) were fitted at 532.0 eV, which is associated with the lattice oxygen in the prepared NFs and expressed O^2−^ ionization state.^34,35^ Moreover, Fig. 1(c) presents the XPS spectra of the core level Cr 2p orbital, which further dissociates into the spin orbitals of Cr 2p_3/2_ and Cr 2p_1/2_, and are located at 578.0 and 588.0 eV, respectively. The spin energy separation of Cr 2p_3/2_ and Cr 2p_1/2_ is 10.0 eV, and can be ascribed to Cr^3+^ in the ZnO/MgO/Cr_2_O_3_ NFs.^36^ Furthermore, the high-resolution XPS spectra of Mg 2p (as in Fig. 1(d)) are located at 50.4 eV, which is a characteristic value for the Mg^2+^ oxidation, as reported earlier.^37,38^

### The structural morphology of the ZnO/MgO/Cr_2_O_3_ NFs analyzed by FESEM

**Fig. 2:**
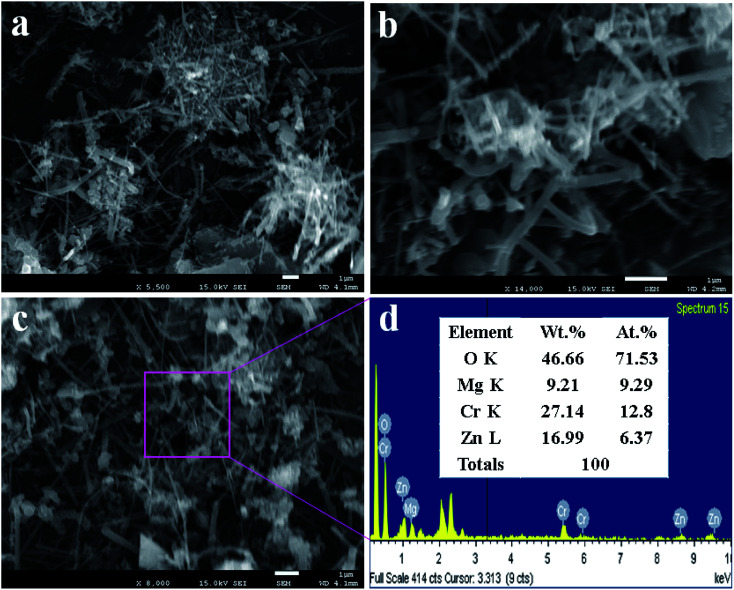
The structural morphology and atomic compositions of the ZnO/MgO/Cr_2_O_3_ NFs obtained by FESEM analysis. (a and b) The low and high magnification FESEM images, (c) image of the EDS-confirmed nanofiber morphology of the ZnO/MgO/Cr_2_O_3_ nanocomposites, and (d) the elemental compositions of ZnO/MgO/Cr_2_O_3_ NFs analyzed by EDS.

In this approach, the lower and higher magnification FESEM images of ZnO/MgO/Cr_2_O_3_ NFs are demonstrate in Fig. 2(a and b). As revealed from Fig. 2(a and b), the prepared nanocomposite exhibited the fibre-like complex morphology. Therefore, it can be concluded that the prepared ternary mixed metal oxide has fiber-shaped morphology with the irregular arrangement. The similar morphology of the nanomaterials with ZnO are reported elsewhere.^39,40^ The average cross-section diameter of the fiber is 68.7 nm, which was obtained in a range of 60.0 to 85 nm. The elemental analysis of ZnO/MgO/Cr_2_O_3_ executed by EDS, as in Fig. 2(c and d) and Fig. 2(c), confirmed the nanofiber morphology of the synthesized ZnO/MgO/Cr_2_O_3_ composites. The elemental analysis obtained from EDS provided evidence of the existence of only O, Zn, Mg, and Cr, and any peak due to impurities was not detected. The elemental composition of the ZnO/MgO/Cr_2_O_3_ NFs is 46.66%, 9.21%, 27.14%, and 16.99% corresponding to O, Mg, Cr and Zn, respectively.

### Optical characterization of ZnO/MgO/Cr_2_O_3_ NFs

**Fig. 3:**
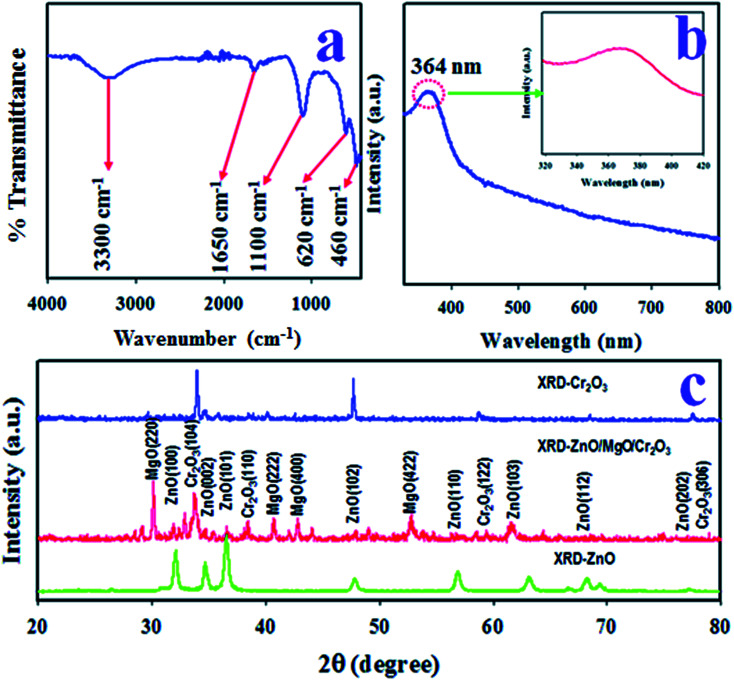
Optical characterization of the ZnO/MgO/Cr_2_O_3_ NFs. (a) FTIR investigation to identify the existing functional groups in the synthesized NFs. (b) Evolution of the UV-Vis adsorption to calculate the optical band gap, and (c) the crystallinity and average grain size obtained from the XPD results.

The existing functional groups of the ZnO/MgO/Cr_2_O_3_ NFs were identified by FTIR spectra, which are shown in Fig. 3(a). The FTIR investigation was executed in the 450–4000 cm^−1^ range, as shown in Fig. 3(a), and the absorption bands recorded at 460 and 620 cm^−1^ correspond to the Zn–O and Mg–O optical stretching vibrations, respectively.^41,42^ Besides this, the observed FTIR peaks at 1100, 1650 and 3300 cm^−1^ were obtained for the stretching vibration of Mg–OH, and the bending and bonding vibration of the OH group, respectively.^43,44^

The photo-absorption of the ZnO/MgO/Cr_2_O_3_ NFs was investigated by applying UV-Vis spectroscopic analysis in the range of 300–800 nm, as shown in Fig. 3(b), which displayed a visible adsorption band at 364 nm due to the transition of valence electrons from the low to high energy levels.^45^ Therefore, the optical band gap of the ZnO/MgO/Cr_2_O_3_ NFs was calculated following eqn (vii), and the obtained bandgap is 3.41 eV:

vii *E*_bg_ (eV) = 1240/*λ*

where *E*_bg_ is the band-gap energy, and *λ* is labeled as the highest absorbed wave-length.

The crystalline phases of the ZnO/MgO/Cr_2_O_3_ NFs were inspected using X-ray powder diffraction (XRD) analysis using a Cu-Kα radiation source at 1.5406 Å in the range of 2*θ* (20°–80°), as illustrated in Fig. 3(c), which consists of the ZnO, MgO, and Cr_2_O_3_ crystalline phases only. As shown in Fig. 3(c), the diffracted peaks of ZnO are (100), (002), (101), (102), (110), (103), (112) and (102) planes, which were identified by JCPDS no. 065-3411 and previous articles on ZnO.^46,47^ Besides this, the crystalline phases of MgO are (220), (222), (400) and (422). These reflected peaks of MgO were identified by JCPDS no. 004-0829 and previous authors.^48,49^ Moreover, several reflected XRD peaks for Cr_2_O_3_ are (104), (110), (122), and (306). These Cr_2_O_3_ phases were found to have very close similarity with JCPDS no. 038-1479 and reported articles.^50,51^ Separately, the powder XRD patterns of ZnO and Cr_2_O_3_ were measured, and are presented in the same figure (Fig. 3(c)). The average grain sizes of the NFs were assessed at the NFs (104) peak using the Scherer equation, as shown in eqn (viii), and were found to be 42.51 nm:^52^

viii *D* = 0.9*λ*/(*β* cos *θ*)

where *λ* is the wavelength of the X-ray radiation (1.5418 Å), and *β* is the full width at half maximum (FWHM) of the peak at diffracted angle *θ*.

### BET analysis

**Fig. 4:**
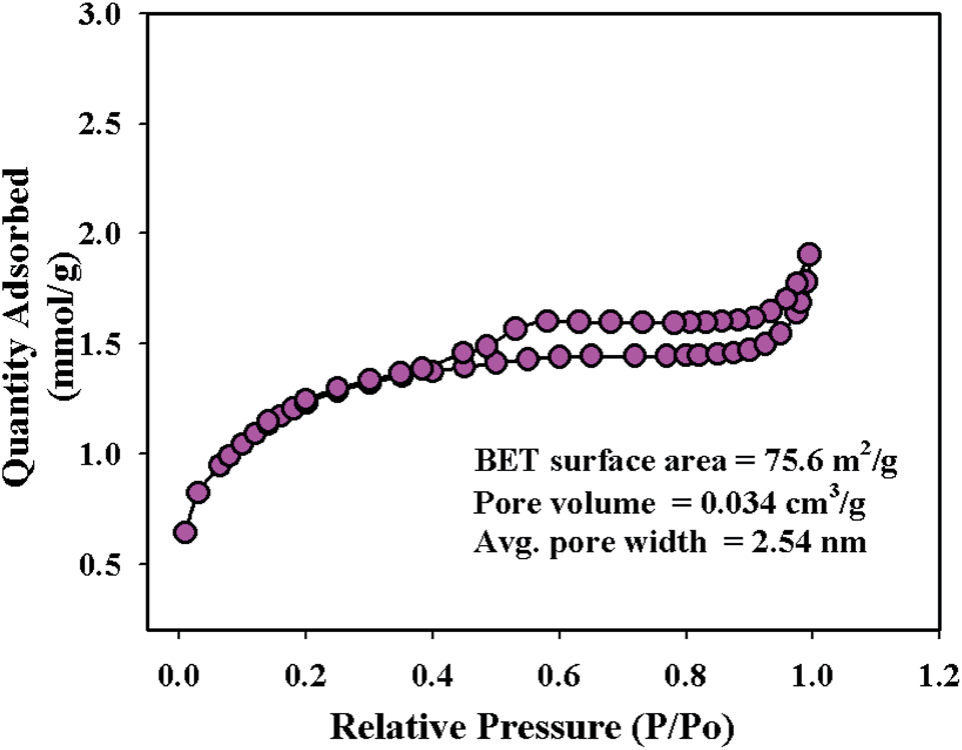
Investigation of BET for ZnO/MgO/Cr_2_O_3_ nanofibers.

To investigate the surface area, pore volume, and pore diameter, BET analysis was performed on the synthesized NFs using the 3Flex analyzer (Micromeritics, USA) at 77.0 K. From the multi-point N_2_ adsorption–desorption isotherm, the specific surface area of the ZnO/MgO/Cr_2_O_3_ NFs was calculated applying BET theory on the linear part of the BET-isotherm, as shown in Fig. 4. As shown in Fig. 4, the BET analysis report of the ZnO/MgO/Cr_2_O_3_ NFs shows the IV-type isotherm containing a hysteresis loop, which represented the mesoporous ordered textural pores. Besides this, the BET report of the ZnO/MgO/Cr_2_O_3_ NFs exhibited a high surface area of 75.6 m^2^ g^−1^, 2.54 nm pore diameter and 0.034 cm^3^ g^−1^ pore volume. The surface area of the ZnO/MgO/Cr_2_O_3_ NFs indicated the case cavities that resulted in the reduction of the reactant chemical precursors using an alkaline medium.

### TEM analysis

**Fig. 5:**
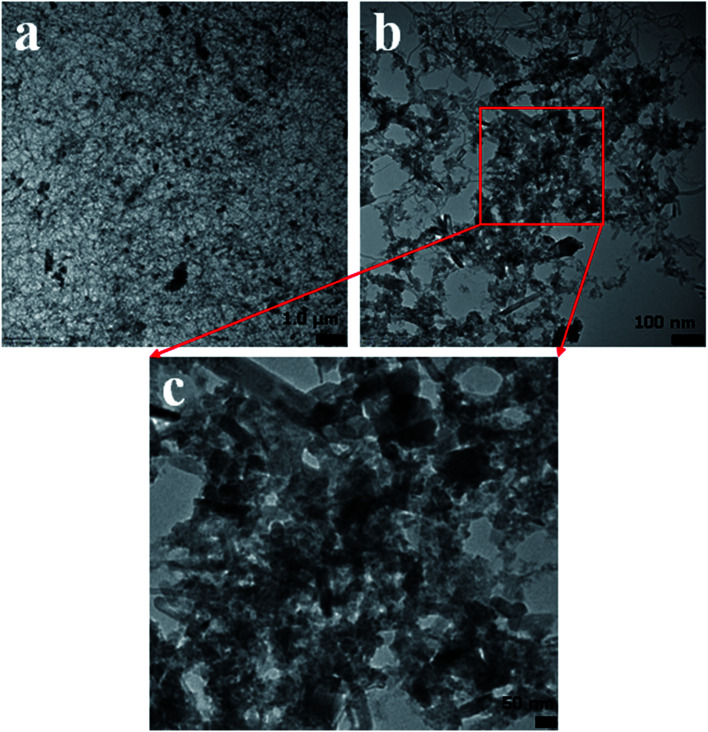
(a–c) TEM analysis from low to high magnification images of the ZnO/MgO/Cr_2_O_3_ nanofibers.

In this approach, the morphology of the ZnO/MgO/Cr_2_O_3_ nanofibers was evaluated by TEM analysis. As observed from Fig. 5, the aggregated fiber-shaped morphology was exhibited in the ternary doped ZnO/MgO/Cr_2_O_3_ materials. The TEM images (Fig. 5(a–c)) show the ZnO/MgO/Cr_2_O_3_ nanofiber aggregated onto the surface of ternary materials, confirming the successful synthesis of the fiber materials of ZnO/MgO/Cr_2_O_3_. Therefore, it is very clear from the TEM images that the doped nanomaterial was assembled with fiber-like shaped morphology, which represents the aggregation as ternary nanostructure materials.

### Optimization and analyses of the ZnO/MgO/Cr_2_O_3_ NFs sensor performances

**Fig. 6:**
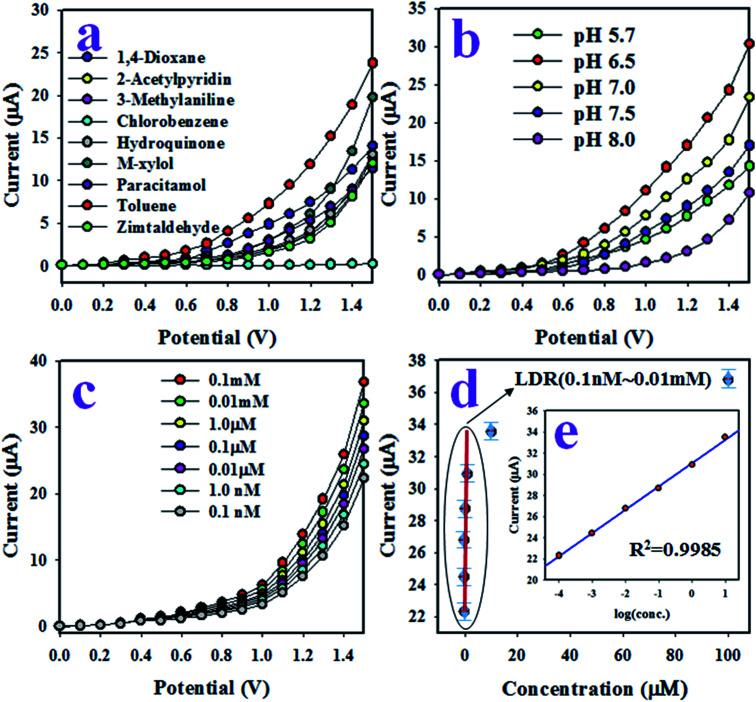
Optimization of the chemical sensor analytical performances of the ZnO/MgO/Cr_2_O_3_ NFs/GCE sensor probe, (a) selectivity assessment with analyte concentration of 0.1 μM, (b) pH optimization at 0.1 μM toluene, (c) electrochemical responses to toluene concentration, and (d) exploration of the calibration curve [inset: log(toluene-conc.) *vs.* current].

The working electrode of the desired toluene sensor probe was constructed with GCE coated by a thin uniform layer of wet-chemically prepared ZnO/MgO/Cr_2_O_3_ NFs. The required binding strength between the thin films of ZnO/MgO/Cr_2_O_3_ NFs and GCE was increased by the addition of Nafion adhesive. Due to the conductive nature of Nafion, it improves the conductance and electron transfer rate of the sensor reported previously.^52,53^ In the electrochemical (*I*–*V*) analysis using the assembled sensor probe, the current density was measured on the surface of a thin film of ZnO/MgO/Cr_2_O_3_ NFs/GCE, and the holding time in the electrometer was set to 1 s as a constant throughout the experiment. To assess the selectivity of the sensor with the ZnO/MgO/Cr_2_O_3_ NFs/GCE electrode, the various environmental toxins with a concentration of 0.1 μM were analyzed at pH 7.0 in a potential range of 0 to +1.5 V. The resultant electrochemical responses of 2-acetylpyridin, M-xylol, 3-methylaniline, zimtaldehyde, 1,4-dioxane, toluene, chlorobenzene, hydroquinone, and paracetamol are presented in Fig. 6(a). Compared to the analyzed chemicals, toluene exhibited the uppermost electrochemical (*I*–*V*) response. According to the mechanism of the reaction (ix)–(xi), electrons are released in the aqueous system due to the oxidation of toluene on the surface of the ZnO/MgO/Cr_2_O_3_ NFs/GCE, which improved and enhanced the current responses against the potential during the electrochemical measurement. As the highest *I*–*V* response is obtained from toluene, it is then considered as the selective analyte for the sensor probe.

It is not possible that the fabricated ZnO/MgO/Cr_2_O_3_ NFs/GCE sensor probe would show the identical electrochemical result towards toluene analyte in the phosphate buffer solution in different pH. Therefore, the pH value optimization is necessary to obtain the maximum electrochemical outcome from the sensor. As a result, the fabricated sensor was subjected to analyze toluene in a wide pH range (5.7–8.0) of buffer solutions. The pH-dependent electrochemical responses of toluene with a concentration of 0.1 μM were studied at applied potentials of 0 to +1.5 V, as illustrated in Fig. 6(b). As shown in Fig. 6(b), the sensor based on the ZnO/MgO/Cr_2_O_3_ NFs/GCE was found to show the highest electrochemical response at pH 6.5 in the analysis of toluene. Thus, pH 6.5 is the optimum condition for the electrochemical oxidation of toluene on the assembled sensor probe.

After that, a series of toluene solutions with varying concentrations ranging from 0.1 mM to 0.1 nM were investigated at a potential of 0 to +1.5 V in the buffer solution of pH 6.5, and the resultant electrochemical responses are shown in Fig. 6(c). As observed in Fig. 6(c), the electrochemical outcomes were amplified with increasing toluene concentration in the sensing buffer solution in a sequence of low to high, and distinguishable from each other. The similar tendency in the *I*–*V* responses have been observed in the detection of various toxins, as reported earlier.^54,55^ To evaluate the analytical parameters, such as sensitivity, LDR, LOD, and LOQ of the projected toluene sensor, a linear relation of current *versus* toluene concentration was plotted, as shown in Fig. 6(d), and known as the calibration curve of the toluene sensor. For this plot, the current data were isolated at a potential of +1.5 V from Fig. 6(c). The sensor sensitivity was calculated from the slope of the calibration curve (0.7551 μA μM^−1^) by considering the active surface area (0.0316 cm^2^) of GCE. The estimated sensitivity was found to be 23.89 μA μM^−1^ cm^−2^. From the inset in Fig. 6(d), the current *versus* the logarithm of the concentration plot was found to be linear (regression co-efficient *R*^2^ = 0.9958) in the range of 0.1 nM to 0.01 mM, which is defined as a linear dynamic range (LDR) of the sensor to toluene detection. Obviously, this LDR is wider. The LOD and LOQ of the toluene sensor based on ZnO/MgO/Cr_2_O_3_ NFs/GCE were computed using a signal/noise (S/N) ratio of 3, and the resultant values were equal to 95.59 ± 1.50 pM and 318.63 ± 2.0 pM, respectively. Thus, the resultant LOD and LOQ values are appreciably lower for toluene detection.

**Scheme 1:**
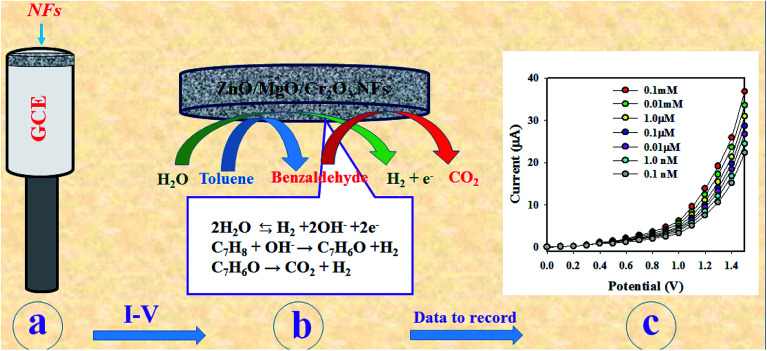
Schematic representation of the sensing mechanism to detect toluene by ZnO/MgO/Cr_2_O_3_ NFs/GCE during electrochemical analysis. (a) The modification process of GCE with NFs, (b) the *I*–*V* detection of toluene, and (c) the resultant data to record at the electrometer.

As observed in Fig. 6(d), the current responses were distributed evenly on a line. Therefore, it provided the evidence for the reliability of this method. Then, it was obvious that the ZnO/MgO/Cr_2_O_3_ NFs/GCE-based electrode could be used to determine the toluene level in buffer solutions in a wide range of concentrations. The proposed oxidation mechanism of toluene in the phosphate buffer system is illustrated in Scheme 1. In the *I*–*V* analysis, toluene is oxidized to generate free electrons, which are responsible for the enhancement of the conductance of the sensing buffer phase. As a result, the intensive electrochemical (*I*–*V*) response was detected. As shown in the reaction (ix)–(xi), the water molecules are adsorbed on the surface of the ZnO/MgO/Cr_2_O_3_ NFs/GCE electrode, and OH^−^, H_2_ and electrons are generated. Subsequently, OH^−^ is reacted with C_7_H_8_ to produce C_6_H_6_O and finally, CO_2_ and H_2_. The similar electrochemical oxidation of toluene has been reported earlier.^56,57^ix
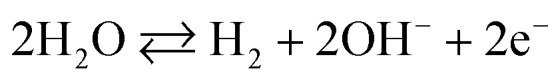

x C_7_H_8_ + OH^−^ → C_7_H_6_O + H_2_

xi C_7_H_6_O → CO_2_ + H_2_

**Fig. 7:**
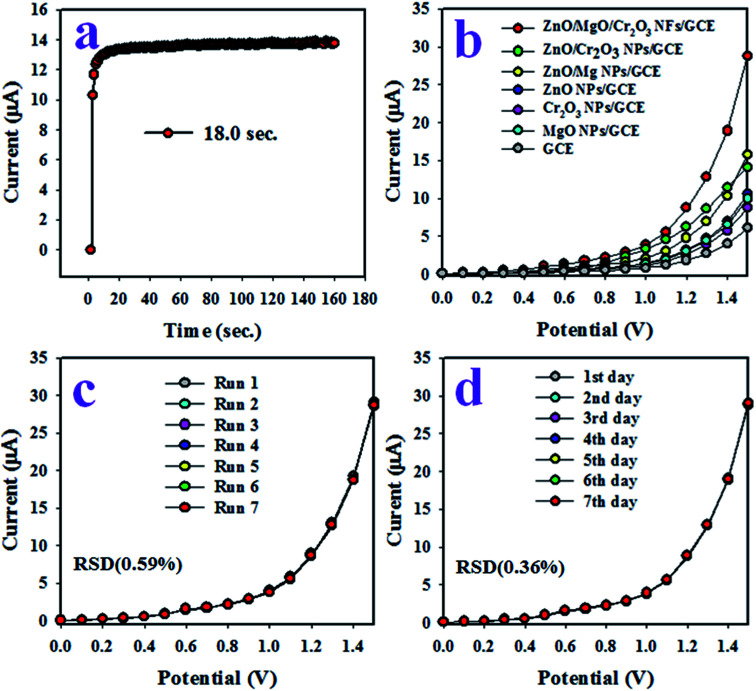
Assessment of the reliability parameters of the toluene sensor with ZnO/MgO/Cr_2_O_3_ NFs/GCE. (a) The assessment of the response time of the toluene sensor at 0.1 μM. (b) The comparison between the electrochemical responses of the sensor with GCE modified by single, binary and ternary metal oxides at 0.01 μM toluene and pH 6.5. (c) The reproducibility parameter tested at 0.1 μM, and (d) the long-time performance ability test of the toluene sensor.

The response time is a tool to evaluate the efficiency of an electrochemical sensor, and is defined as the time required to complete an electrochemical analysis of an analyte. Therefore, the response time of the toluene sensor with ZnO/MgO/Cr_2_O_3_ NFs/GCE was tested with 0.1 μM toluene at a potential of 0 to +1.5 V in the buffer solution, and the result is shown in Fig. 7(a). As in Fig. 6(a), the response time was achieved at 18.0 s, which is appreciable and provides the information about the high efficiency of the toluene sensor probe. Fig. 7(b) shows the electrochemical responses of the various modified GCE with ZnO/MgO/Cr_2_O_3_ NFs, ZnO/MgO NPs, ZnO/Cr_2_O_3_ NPs, ZnO NPs, MgO NPs and Cr_2_O_3_ NPs in identical conditions (0.01 μM toluene analyte concentration; applied potential range of 0 to +1.5 V; phosphate butter phase at pH 6.5. The GCE is modified by a ternary mixture of ZnO/MgO/Cr_2_O_3_ NFs, and shows the superlative *I*–*V* response compared to the binary ZnO/MgO NPs, ZnO/Cr_2_O_3_ NPs, and single ZnO NPs, MgO NPs and Cr_2_O_3_ NPs. It occurred owing to the combinational effects of the co-doped ternary metal oxides. The other cause is that the conductivity of the mixed metal oxides is increased compared to the single or binary NPs. As a result, the ZnO/MgO/Cr_2_O_3_ NFs showed the highest electrochemical activity toward toluene (analyte), as shown in Fig. 7(b). The reproducibility performance of the toluene sensor was tested in 0.01 μM toluene in the buffer at pH 6.5 (potential range: 0 to +1.5 V). As observed in Fig. 7(c), the replicated seven runs are practically undistinguishable in identical conditions, which confirmed the evidence of the consistency of the toluene chemical sensor with the ZnO/MgO/Cr_2_O_3_ NFs fabricated sensor probe. The magnitude of the electrochemical responses of the reproducibility test was not altered even after washing the electrode with PBS buffer in each run. The precision of the current data of the reproducibility test at a potential of +1.5 V was calculated, and found to be ±0.59% in the form of a relative standard deviation (RSD). It provided evidence of the high precision of reproducibility of the selective toluene electrochemical sensor. The tests under the identical conditions of concentration of target toluene, pH of the medium, and applied potential were executed for reproducibility. They were performed for an extended period in consecutive seven days, and are presented in Fig. 6(d). The relative standard deviation (RSD) of the current data of these tests at +1.5 V was calculated, and it was found as ±1.0%. In conclusion, the toluene sensor with ZnO/MgO/Cr_2_O_3_ NFs/GCE showed long-term stability with high precision in sensing performance. It is shown in Fig. 5(c) that the current density measured in the buffer system is directly proportional to the corresponding concentration of toluene.

Thus, the amplified *I*–*V* responses were observed with increasing toluene concentration. At the very beginning of the toluene detection performance, the surface coverage with the adsorption of a few numbers of analyte (toluene) molecules was smaller. In addition, the oxidation reaction of toluene on the surface of the working electrode was started progressively. With the enhancement of toluene molecules, the surface reaction and coverage both increased gradually and approached its equilibrium saturation state. Furthermore, the enhancement of the toluene concentration in the sensing buffer medium, a steady-state current density and the reaction rate were attained. Such steady-state equilibrium data are shown in Fig. 6(d), where the current data are scattered on a line. Therefore, the projected toluene sensor with ZnO/MgO/Cr_2_O_3_ NFs/GCE was able to detect and quantify the targeted analyte (toluene) in the real-field of its application. Previously, it has been projected that the response time of the toluene chemical sensor is around 18.0 s, and some reserve time of 20.0 s is needed to attain the steady-state equilibrium *I*–*V* response, as shown in Fig. 7(a). As demonstrated in Fig. S1 and S2 in the ESI section (ESM), Fig. S1(a)† presents the three individual *I*–*V* responses of toluene, M-xylol and 1,4-dioxane, respectively, which are completely distinguishable. On the other hand, Fig. S1(b)† illustrates the four *I*–*V* responses, such as ‘toluene’, ‘toluene & M-xylol’, ‘toluene & 1,4-dioxane’ and ‘toluene, M-xylol & 1,4-dioxane’, which are indistinguishable. Therefore, these tests provided evidence that the ZnO/MgO/Cr_2_O_3_ NFs/GCE chemical sensor is selective to toluene, and there is no measurable interference effect in the presence of another toxin in the measuring system. Therefore, the toluene chemical sensor with ZnO/MgO/Cr_2_O_3_ NFs/GCE is a promising sensor to detect toluene selectively, reliably and precisely in the real environment of its application. Nafion has no significant role in the sensing of the target analyte with the ZnO/MgO/Cr_2_O_3_ NFs/GCE sensor probe in the *I*–*V* measurement. In addition, a control experiment was executed with the various modification electrodes (Bare GCE, Nafion/GCE, and ZnO/MgO/Cr_2_O_3_ NFs/GCE), which is included in the ESM (Fig. S2†). There are no significant changes in the electrochemical signal without and with the Nafion binder during the detection of the target analyte. Here, Nafion is used as a chemical glue to stick the nanofibers onto the GCE. The reliability and stability of the fabricated ZnO/MgO/Cr_2_O_3_ NFs were studied, and are included in the ESI section (Fig. S3†). The *I*–*V* experiment was carried out with the same ZnO/MgO/Cr_2_O_3_ NFs-modified GCE electrode under identical conditions in seven consecutive days. The electrochemical responses were observed with similar results for the detection of the target toluene analyte, and are presented in Fig. S3a.† So, after seven days, the electrochemical response was found to be almost the same as the previous results. On the other hand, the stability of the ZnO/MgO/Cr_2_O_3_ NFs-fabricated GCE was also evaluated in the ferrocyanide couples using voltammograms under identical conditions. It was observed that the fabricated ZnO/MgO/Cr_2_O_3_ NFs/GCE sensor probe showed almost the same response after 20 cycles, which is presented in Fig. S3b.† Therefore, it is concluded that the fabricated ZnO/MgO/Cr_2_O_3_ NFs/GCE sensor probe is very reliable, as well as stable, even after several cycles and days. The ZnO/MgO/Cr_2_O_3_ NFs as an electron mediator can provide a satisfactory nano-environment due to its high crystalline phases with the average molecular size of 42.51 nm, which can offer the successful detection of toluene in the phosphate buffer phase. The synthesized nanofiber of ZnO/MgO/Cr_2_O_3_ also exhibited the average band-gap energy of 3.41 eV, which facilitated the high electron transfer from the active sites of the NFs toward the GCE. As a result, the assembled sensor established a good electron communication to detect toluene in the buffer solution in the electrochemical (*I*–*V*) approach. In Table 1,^9,58,59^ the sensing parameters of the formerly studied electrochemical sensor based on different nanomaterial matrices are illustrated. Among these, the tested toluene sensor based on ZnO/MgO/Cr_2_O_3_ NFs/GCE showed appreciable performances.

**Table 1:** The comparison of the analytical performances of the proposed toluene sensor with similar works

Electrode	Analyte	DL^a^	LDR^b^	Sensitivity	Ref.
ZnO/Yb_2_O_3_NSs/GCE	4-Aminophenol	94 pM	1.0 nM–0.1 mM	5.06 μA μM^−1^ cm^−2^	9
MnCo_*x*_O_*y*_ NPs/GCE	3,4-Diaminotoluene	0.26 pM	1.0 pM–1.0 μM	0.37 μA μM^−1^ cm^−2^	59
FeO/CdO NCs/GCE	1,2-Dichlorobenzene	72.73 nM	0.089 nM–8.9 mM	1.31 μA μM^−1^ cm^−2^	60
ZnO/MgO/Cr_2_O_3_ NFs/GCE	Toluene	95.59 pM	0.1 nM–0.01 mM	23.89 μA μM^−1^ cm^−2^	This report

a DL (detection limit).

b LDR (linear dynamic range), pM (picomolar), mM (millimolar).

### Analyses of real environmental samples

The standard recovery test of the proposed toluene chemical sensor probe based on ZnO/MgO/Cr_2_O_3_ NFs/GCE was performed by using an electrochemical (*I*–*V*) approach. To investigate the analysis, a known concentration of the target toluene solution was added to the collected real samples. The resultant data were systematically investigated to measure the concentration of the target toluene again, which was calculated as % recovery. In this analysis, the environmental samples were collected and extracted from different sources, such as seawater, PC-baby bottle, PVC-food packaging bag, PVC-water bottle, and waste effluent from the industrial area. The resulting data are summarized in Table 2. The results for the validation of the fabricated sensor were found to be quite acceptable.

**Table 2:** The electrochemical analysis of real samples with ZnO/MgO/Cr_2_O_3_ NFs/GCE probe applying the electrochemical approach

Real samples	Added tol conc. (μM)	Measured toluene conc. by ZnO/MgO/Cr_2_O_3_ NFs/GCE^a^ (μM)			Average recovery^b^ (%)	RSD^c^ (*n* = 3) (%)
		*R*_1_	*R*_2_	*R*_3_		
Industrial effluent	0.01000	0.009494	0.009405	0.009573	94.91	0.89
PC-baby bottle	0.01000	0.009832	0.01001	0.009827	98.89	1.05
PVC-water bottle	0.01000	0.009783	0.009589	0.009613	96.62	1.09
PVC-food packaging bag	0.01000	0.009593	0.009571	0.009680	96.15	0.60
Sea water	0.01000	0.009350	0.009047	0.009192	91.96	1.65

a Mean of three repeated determination (signal to noise ratio 3) with ZnO/MgO/Cr_2_O_3_ NFs/GCE.

b Concentration of toluene determined/concentration taken (unit: μM).

c Relative standard deviation value indicates precision among three repeated measurements (*R*_1_, *R*_2_, *R*_3_).

## Conclusion

The selective toluene electrochemical sensor was prepared by GCE coated with calcined ZnO/MgO/Cr_2_O_3_ NFs as the layer of thin-film using 5% Nafion adhesive. The proposed sensor was found to be reliable in determining the toluene level in PBS (buffer solution) medium. It shows the excellent responses in terms of the sensitivity, DOL, LOQ, LDR, reproducibility, and stability. Besides this, it is a successful sensor in the detection of toluene in real wastewater polluted samples by the recovery method. Therefore, this research work might be a noble approach for the development of a future chemical sensor probe to identify unsafe toxins in the environmental safety and health care sectors in broad scales.

## Conflicts of interest

There are no conflicts to declare.

## Supplementary Material

RA-010-D0RA08577D-s001
