# Supplementary material for: An alternative electrochemical approach for toluene detection with ZnO/MgO/Cr2O3 nanofibers on a glassy carbon electrode for environmental monitoring
Source: RSC Adv. 2020 Dec 17;10(73):44641–53. doi: 10.1039/d0ra08577d (PMC9058479; doi:10.1039/d0ra08577d)
Supplement: RA-010-D0RA08577D-s001 [file RA-010-D0RA08577D-s001.pdf]

Electronic Supplementary Material (ESI) for RSC Advances.
This journal is © The Royal Society of Chemistry 2020

Supporting information

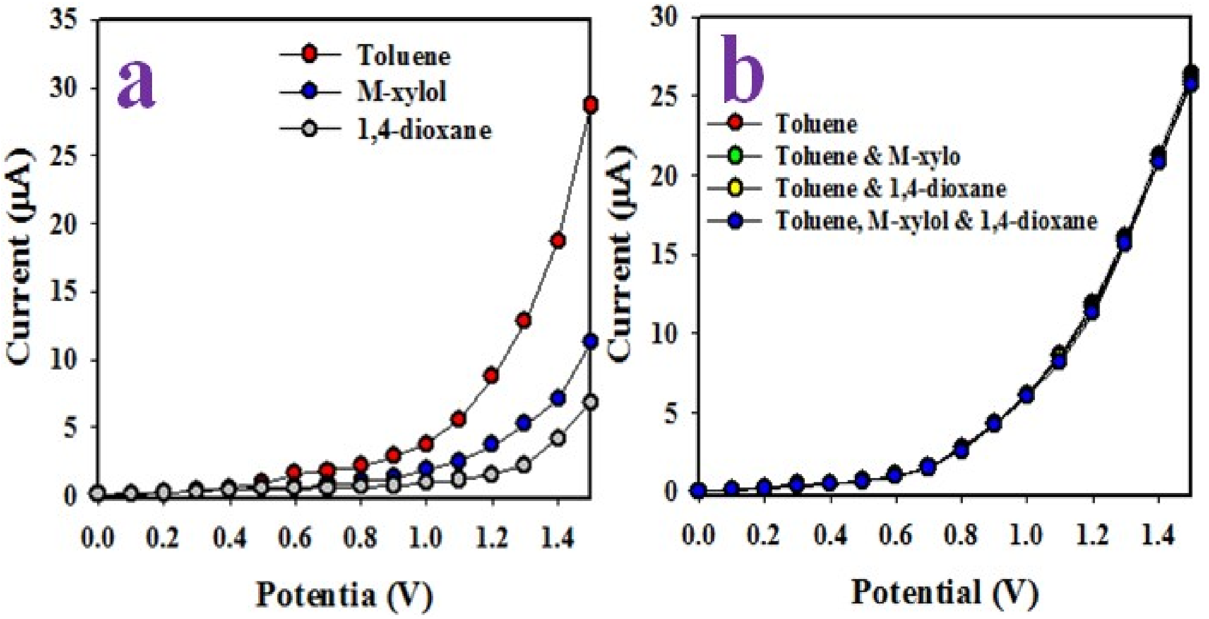

Fig. S1. Optimization of sensor. (a) Comparison studies between electrochemical responses of toluene, M-xylol, and 1,4-dioxane compounds based on $ZnO/MgO/Cr_2O_3$ NFs/nafion/GCE sensor probe, (b) Interference effect in electrochemical response of toluene chemical sensor.

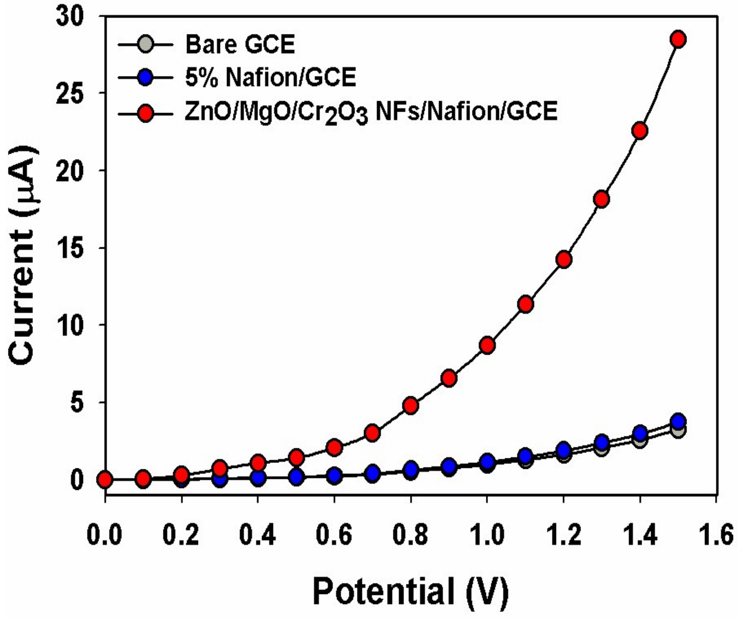

Fig. S2. Control experiment with various electrode modification (Bare GCE, Nafion/GCE and $ZnO/MgO/Cr_2O_3$ NFs/Nafion/GCE) in the identical conditions. Analytes concentration 0.1 μM; Potential range: 0 to 1.5 V

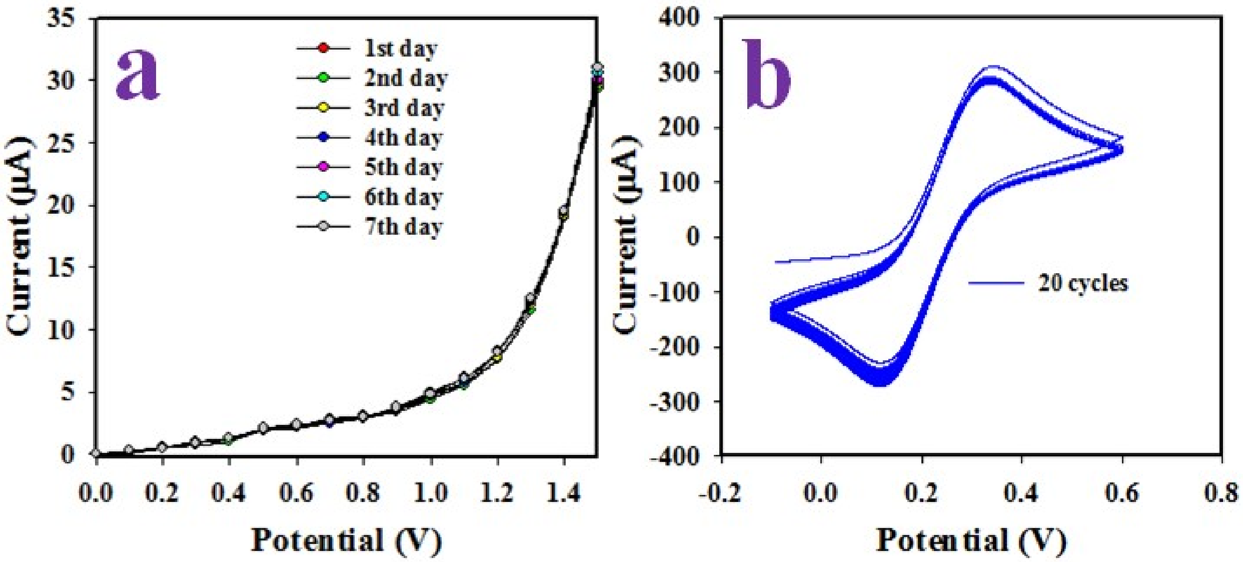

Fig. S3. Reliability and stability of the ZnO/MgO/Cr_2O_3 NFs/Nafion/GCE sensor probe. (a) I-V responses with the same modified electrode in different days for reliability study and (b) Voltammograms (20 cycles) of the fabricated electrode in the ferrocyanide couple for stability study.
